# Dementia and Challenging Behaviors in Gerontological Centers. A Case Report

**DOI:** 10.3390/geriatrics4010015

**Published:** 2019-01-22

**Authors:** Romina Mouriz, José Caamaño Ponte, Laura García Tuñas, Carlos Dosil, David Facal

**Affiliations:** 1 Gerontological Therapeutic Complex “A Veiga”, Serge Lucense, Pobra de San Xiao, Láncara, 27360 Lugo, Spain; rominamouriz@hotmail.com (R.M.); caamajos@hotmail.com (J.C.P.); serge@serge.es (L.G.T.); carlos.dosil@usc.es (C.D.); 2 Department of Developmental Psychology, University of Santiago de Compostel, Santiago de Compostela, 15782 Galicia, Spain

**Keywords:** cognitive impairment, challenging behaviors, Lewy bodies dementia

## Abstract

Among the main challenges in geriatric and gerontological centers, we consider, central, the individualized attention to those elderly persons with challenging behaviors, to the extent that it is possible to design preventive strategies that delay cognitive deterioration and minimize consequences of behavior disorders. The first step will be to develop the correct interpretation of symptoms and deficits as a guarantee of a correct diagnosis which, in addition to not always being easy, has to be adapted to the progression of the disease. We present the case of a 68-year-old institutionalized individual, with an initial diagnosis of diffuse Lewy bodies dementia, analyzing his cognitive and behavioral evolution, and the pharmacological and non-pharmacological approach to the case.

## 1. Introduction

Geriatric and gerontological institutions constitute an essential element in the care of individuals in situations of age-related fragility and dependence. One of the main challenges of these institutions is having to deal with the constant increase in the number of people that develop challenging behaviors (CBs) related to cognitive decline and dementia. These CBs are not only crucial in the evolution of the disease, but also in the therapeutic approach, in establishing relationships between the staff and users and, ultimately, in the functioning of the institution.

Dementias constitute a problem of global magnitude. Thus, in the year 2015, there were 47 million people with diagnosed dementia [1]. Countries, such as France and Italy, have registered more than 1.1 million people with a diagnosis, whereas in Spain the estimated total is between 600,000 and 800,000 persons affected [2]. Although LDB are less common (11%) than Alzheimer’s disease (AD) (31%) or vascular dementia (VD) (22%) [3], it occurs sufficiently often to be seen with frequency in clinical practice and care institutions. In this type of dementia, the behavioral symptoms can be more premature and more intense. It seems that “mixed” forms are more common than “pure” forms, particularly for AD and LDB, according to the Neuropathology Group of the Medical Research Council Cognitive Function and Ageing Study [4]. In addition, if we observe the diagnosis guidelines for the different types of dementia, the behavioral symptoms are presented with subtle differences in the beginning and throughout the evolution of the disease [5,6]. In the case of the LDB, the presence of recurrent visual hallucinations, typically well-formed and with a high level of detail, is considered a key symptom of the disease [7].

In the context of a degenerative disease that involves the progression of cognitive decline and behavioral manifestations and an increasing degree of difficulty in controlling these behaviors, a comprehensive approach includes in those patients with severe behavioral problems the institutionalization in specialized centers. In these patients are central the management of behavioral disorders, their correct identification and, if possible, the development of novel therapeutic strategies.

In the context of dementia, these behaviors have been called behavioral and psychological symptoms of dementia (BPSD), although we believe that, at an institutional level, the use of the term CB is clearer, since it reflects the consequences of functional conducts, and it facilitates the elaboration of strategies for multidimensional intervention [8,9]. It must be noted that not all individuals with psychological disorders present a dementia diagnosis. CBs are alterations in the mood, thoughts, perceptions, and conducts of the elderly that are perceived as “not rational” and that limit the efficacy of the care, calling into question the norms of the institution itself [9]. CBs can be divided into four categories [8]: (1) agitation, including lack of inhibition, wandering, repeated calls or questions, and difficulty regarding personal hygiene; (2) manifestations of a difficult character and/or mood disorders; (3) excess of demanding behaviors and/or solicitous of activity; (4) apathy and tendency to be immobile. From a pragmatic perspective, CBs are heterogeneous conducts with a variable prevalence and characterized by being very distorting. The therapeutic approach usually consists of the use of psychotropic drugs of different subgroups and with arguable efficacy, often presenting pharmacological antagonisms or notable adverse effects on the central (anticholinergic action, sedation, extrapyramidal symptoms) and the peripheral nervous system (hypotension, bradycardia, or arrhythmia). Other factors that have a strong impact on CBs are environmental factors, including the architecture of the institution itself, the existence of hostile or friendly spaces, and social aspects, such as the adaptation of expectations by individuals, families, and professionals to the organizational strategies [10].

## 2. Case

### 2.1. Personal History

The subject of the study was a 68-year-old male, left-handed and a native of a rural area in Lugo (Galicia, NW Spain). He was single and used to live with his mother until she passed away; he then lived alone until 2014, when he was admitted to a geriatric center close to his town. He has two brothers, a basic level of education, and his profession was agricultural worker. 

### 2.2. Ethical Standards

The study was conducted in accordance with the Declaration of Helsinki. Written informed consent was obtained from the participant and the legal responsible.

### 2.3. Medical History

According to the medical records, the patient presented arterial hypertension, hypertensive heart disease, paroxysmal atrial fibrillation, mild kidney failure, diabetes mellitus—type II, cholecystitis, confusional syndrome, dyslipidemia, anxiety disorder, inguinal hernia, and diffuse Lewy bodies dementia (LBD). The patient did not present toxic habits.

### 2.4. History of the Disease

The documentation provided by the family in reference to the development, prior to being admitted into our gerontological center, shows an oscillating clinical course, with crisis and a negative development, with difficulties for a neuropsychiatric differential diagnosis and a clear therapeutic approach. In particular, the medical records from December 2014 show admissions in August and November of the same year in relation to “disorientation and behavioral disturbances evolving during these months”, with a hypothetic diagnose of VD vs. Parkinson disease (PD) and treatment with levodopa/carbidopa. During the same month, he showed extreme agitation and escaped from the geriatric center where he used to live. He was, therefore, examined by the Emergency Service of the Hospital Universitario de Lugo (HULA). The examination upon admission suggests that “he is disoriented in time and space, person oriented, hardly cooperative, he employs circumlocutions with partially incoherent speech, irritable, shaky, anxious and presents visual hallucinations”. Admitted in the Psychiatry Service of the same hospital, the examination shows “amnesia about his stay in the institution and about his hospital admission, incoherent speech. Lack of data about hallucinatory—delirious symptomatology. Sleeping and eating disorders. General sense of wellbeing, he ignores the reason for his admission. Unawareness of the disease”. Regarding the evolution in the hospital, the level of consciousness tends to fluctuate, with episodes of visual hallucinations without emotional response, relating situations and images with a high level of detail during periods of a few short minutes. These symptoms are presented without structured delusional ideation. 

Following pharmacological adjustment, there is a gradual improvement of the visual hallucinations, but the patient still shows episodes of isolated irritability related to brief confusional states. The patient’s sleep pattern improves, but the noticeable cognitive impairment and functional dependency persists. Following pharmacological adjustment with quetiapine, 150 mg/day, there is a notable worsening of the symptoms of confusion and treatment is therefore suspended, maintaining olanzapine and tiapride. 

The patient remains at the hospital for 24 days and, during that time, he is explored using additional tests. New pharmacological adjustment is done with the aim of decreasing the fluctuations in the level of consciousness, a remission of the visual hallucinations, and a decline in behavior alterations. He is referred to the primary-care center for follow-up, and it is recommended that he be re-admitted to a geriatric center, with a diagnosis of LDB in the presence of a progressive cognitive decline, consciousness fluctuations, visual hallucinations, and parkinsonism. Treatment using olanzapine is suggested, 5 mg/night/24 h, and tiapride, 100 mg/8 h. Lorazepam 1 mg is also prescribed, without signs of anxiety or insomnia. The following medications are kept: omeprazole 20, simvastatin 20, acetylsalicylic acid 100, bisoprolol 2.5, doxazosin 4, and insulin Lantus.

### 2.5. Supplementary Tests

Consultation with the Neurology Service for physical examination, concludes that there are signs of rigid-akinetic parkinsonism and risk of falling. Blood test: dementia screening showed normal parameters in hematimetry and biochemistry. Human immunodeficiency virus (HIV) serology, syphilis, hepatitis B & C are negative. Electroencephalography with frequency analysis shows a “daytime NREM I wakefulness and sleep pattern with well-configured, symmetric, normoreactive, with no appreciable anomalies, neither under resting conditions nor during the activations performed. Signs of drowsiness”. Magnetic resonance imaging (MRI) scan includes “exploration performed in sagittal and axial planes in habitual sequences. There is a small spot of increased signal in T2 and flair in the subcortical white matter compatible with small vessel ischemic pathology. No recent ischemic pathology or other significant findings are evident”.

### 2.6. Follow-Up during 2015

The patient is institutionalized and followed by external consultations with the Psychiatry Service, making adjustments to pharmacological treatment in accordance with the symptomatology. He visits the HULA emergency department as a result of behavioral changes and aggressive behaviors. At that time, he was treated with lorazepam 1 mg/24 h, olanzapine 15 mg/24 h, and clonazepam 1.5 mg/24 h. Tiapride solution is added in case of agitation. 

Re-admitted to the Psychiatry Service due to an episode of agitation and aggression in the institutional environment. At that time, he was treated with rivastigmine 4.6 mg/24 h, clonazepam 4.5 mg/24 h, and clozapine 100 mg/24 h, given the therapeutic failure and the apparent paradoxical response with quetiapine, olanzapine, risperidone, haloperidol, and tiapride, in addition to the treatment for heart diseases and diabetes. During the hospitalization, the patient lacks awareness of the disease. He is restless, requiring physical restrictions and also hypoprosexic. No alterations are detected in the course or content of thought, although his spontaneous speech is not very fluid and is of a repetitive nature. 

The pharmacological adjustment with rivastigmine 9.5 mg/24 h and the decrease in clonazepam due to somnolence, associated with clozapine up to 150 mg/24 h, seems to partially improve the BPSD, although the presence of low-grade fever and leukocytosis suggest the progressive withdrawal of clozapine. Gabapentin 400 mg/24 h is introduced in order to mitigate the psychomotor restlessness and behavioral alterations, with an excellent response, leading to a referral to the geriatric center where he remained until his transfer to the current gerontological center. On the day of his referral, the treatment continued with rivastigmine 9.5 mg/24 h, lorazepam 3 mg/24 h, trazodone 100 mg/24 h, clonazepam 1 mg/24 h, and gabapentin 400 mg/24 h. 

During the following 6 months, there are oscillations in his cognitive functions and in his CBs, presenting nocturnal agitation, wandering, and disorganized motor behaviors, hostility, insomnia, anxiety, as well as hyperorality, constant verbalization related to food and sexual disinhibition. This symptomatology demands modifications in the pharmacological treatment with variable responses. At the end of November, he is evaluated by the Psychiatry Service, which prescribe rivastigmine 9.5 mg/24 h, trazodone 100 mg/24 h, clonazepam 1 mg/24 h, olanzapine 10 mg/42 h, and quetiapine 100 mg/24 h, withdrawing lorazepam and gabapentin.

## 3. Management of Challenging Behaviors in the Gerontological Centre

### 3.1. Methodology

From 2016, the patient was included in the Comprehensive-Dementia Care Program at the gerontological center, with a methodology that consists of a longitudinal clinical and neuropsychological evaluation, psycho-pharmacological optimization, and behavioral control in adapted spaces. Compared to the former geriatric center, the gerontological center included a more multidisciplinary and comprehensive approach. The dementia care program is carried out in two rooms with a capacity for 30 residents, a friendly atmosphere, a reduction in the rotation of the caretaker staff, and includes occupational therapy supporting autonomy in everyday activities. A protocol consisting of the exploration of clinical and cognitive assessment was carried out using the following protocol: Spanish versions of the Mini-Mental State Examination (MMSE), Severe Mini-Mental State Examination (SMMSE), Cornell’s Scale for Depression in Dementia (CSDD), Neuropsychiatric Inventory (NPI-NH), Cohen-Mansfield Agitation Inventory (CMAI), a scale for apathy measurement in institutionalized dementia patients (APADEM-NH), and the Clinical Dementia Rating (CDR), conducted within a 12-month follow-up [11,12,13].

### 3.2. Results

During the first months in the gerontological center, the clinical examination showed CBs, including general symptoms of agitation, persistent insomnia, anxiety, continuous demand for attention, obsessive ideations, repetitive behaviors such as grabbing people, disorganized behaviors, rigidity, hyperorality, and sexual disinhibition. All these CBs led to a change of care program within the gerontological center. The extrapyramidal symptoms persisted, while the blood tests were shown within the reference parameters, except the hematimetry, that suggested a diagnosis of iron deficiency anemia. The results of the evolutionary test results are shown in Table 1 and Table 2. MMSE scores in Table 2 were used to orient the diagnosis, since the syndrome was eminently not cognitive. 

### 3.3. Therapeutic Procedure

The therapeutic procedure was based on two main points, the optimization of the pharmacological treatment (Table 1) and a non-pharmacological approach, based on the inclusion in a space adapted to the objective needs of the person and to his CBs. Regarding the pharmacological treatment, the increase of ideation and obsessive behaviors in the month of January 2016, in addition to the previous therapeutic failures with clonazepam and bromazepam, led to initiation of the treatment with valproate sodium (1000 mg/24 h), potassium clorazepate (80 mg/24 h), and fluvoxamine (100 mg/24 h), avoiding lorazepam, bromazepam, trazodone, quetiapine, and olanzapine. The initial sedation was controlled by progressively reducing the dose of clorazepate to 15 mg/24 h.

In addition, the non-pharmacological approach was based in the modification of the space in which the patient was located, within the Comprehensive-Dementia Care Program at the center in a specific care environment. According to the behavioral characteristics of the patient, the inclusion in the dementia-care program was focused in the potentiation of their basic skills for activities of daily living (i.e., those related to autonomy in eating behaviors). The unit was environmentally more controlled, with special focus in controlling distractor stimuli and sudden changes in the environment that trigger CBs. The care staff was informed and trained in the CBs presented by the patients; in this case, the staff was informed about the behavioral and functional impairments, and trained about fluctuations and social responses.

After these changes, the patient remained cognitive and behaviorally stable, self-oriented, and space-oriented, although not time-oriented, with adequate levels of attention, with partial apathy, daytime sleepiness, coherent speech, although occasionally repetitive in regard to food and sexual topics. Extrapyramidal symptomatology remained, with predominance of rigidity but without negative evolution. He presented two episodes of respiratory infection, was treated in the gerontological center, and hospitalized twice for acute gastroenteritis and bronchoaspiration, with good response to antibiotic therapy and dietary changes, but without major cognitive or behavioral consequences. The last analysis showed total valproate levels in the therapeutic range, 65.0 (50–125 μg/mL), hematimetry with slight signs of iron-deficiency anemia under treatment with ferrous glycine sulfate, adequate glycemic control, nutritional parameters, and renal and hepatic functions. Psychopharmacological treatment with rivastigmine, clorazepate, valproate, and fluvoxamine was maintained (Table 1).

## 4. Discussion

In this case study, there were three areas of deliberation; the diagnostic certainty; the typification of BPSD and, finally, the pharmacological and non-pharmacological approaches of the CBs.

### 4.1. Diagnostic Certainty

The four main types of dementia (AD, VD, LDB, and PD with dementia –PDD-) share two basic clinical criteria: the cognitive impairment and the presence of BPSD, with variability of onset and types. In their evolution, dementias tend to be mixed, presenting lesions characteristic of each type coexisting in each patient. Thus, Lewy bodies have been observed in the black substance in cases diagnosed with AD and VD, however, it is more common to observe them in dementia associated with PD and in LDB itself, with both being considered in the same group. The clinical characteristics of LDB and PDD include fluctuating cognitive impairment, detailed and recurrent visual hallucinations, in addition to the extrapyramidal symptoms characteristic of PD. However, consensus suggests that the diagnosis of PDD should be made whenever the extrapyramidal symptoms are prior to the cognitive deficit, whereas cognitive and motor deficits coexist in the diagnosis of LDB over time [7,14]. Golimstok [15] explains LDB as a two-stage syndrome, with a prodromal stage, in which there are signs and symptoms of the disease without dementia criteria, and a second stage in which the dementia criteria are present. The prodromal phase would be characterized by mild cognitive impairment without evidence of amnesia; BPSD, such as visual hallucinations preceding the cognitive impairment, accompanied or not by anxiety and depression (considered as risk factors); sleep disturbances, parasomnias, and rapid eye movements of varying type and intensity, with daytime somnolence; dysfunction of the autonomic nervous system with orthostatic hypotension that causes falls, urinary incontinence, gastrointestinal dysfunction, and constipation; extrapyramidal signs, such as tremor of rest and action and, very importantly, olfactory dysfunction, with anosmia or hyposmia that can be associated with rapid eye movements and precede any other symptom. The dementia phase would involve the presence of multiple and progressive cognitive impairment (attention, executive function, visuospatial memory), with oscillating symptoms of confusion and acute visual hallucinations, extrapyramidal symptoms, and progression of sleep–wake rhythm disturbances. In some cases, structured delusions of varied themes and progression with auditory and kinesthetic hallucinations are presented.

Although we believe that the patient fulfills an important part of the diagnostic criteria for LDB [7,14,15], including fluctuating cognitive impairment, visual hallucinations, parkinsonism, dysautonomia, and sleep disorders, cognitive evolution could question the diagnosis of dementia because different cognitive areas have improved. Improvement in cognitive function are evidenced in MMSE [16] scores, with the last score (October 2018) being 25 (0–35), equivalent to a slight deficit, after 3 years of follow-up (Table 1 and Table 2). A more comprehensive and specific neuropsychological exploration could have been more exact, using instruments such as the Lewy Body Composite Risk Score (LBCRS) [17], or the Stroop test, from the beginning. Similarly, the inclusion of biomarkers in the assessment, such as dopamine transporter investigation or MIBG, would have helped in a more precise diagnosis. 

The MRI showing vascular injury, the presence of obsessive and depressive symptoms and the adaptation and good therapeutic response to the introduction of valproate and fluvoxamine, could suggest a differential diagnosis with a bipolar disorder associated with PD.

### 4.2. Typification of CBs

The CBs are the main cause of institutionalization in our context, since they question the family and social functioning. These heterogeneous behaviors have been well-characterized in multiple studies. CBs includes psychological disorders, such as delusions, hallucinations, depression, anxiety, apathy, or sleep disorders, and particularly distorting behavior such as agitation, physical, and verbal aggression, catastrophic reactions, negativism, intrusiveness, wandering, and disinhibition [18,19,20,21]. These behaviors require an integral approach in terms of identifying triggers and reinforcements, optimizing psychopharmacological treatment and designing comprehensive intervention programs involving the person, the environment, and the staff [8,22,23].

According to Moniz-Cook et al., we consider as CBs those alterations in the state of mind, thoughts, perceptions, and behaviors that are perceived as “unreasonable”, and that question the norms and the operation of the geriatric and gerontological centers [23]. The management of CBs requires an individualized, personalized approach, but, at the same time, a systematic and comprehensive care model. The careful observation and deep knowledge of the patients in their different dimensions (cognitive and behavioral, but also sensorial, affective, social, communicative, occupational, and so on) becomes fundamental in the management of these types of behaviors, especially in a gerontological context of formal care and institutionalization. In this regard, non-pharmacological treatments have to be chosen to fit the patient’s past identity, preferences, and abilities [22]. 

In the case presented, different CBs are present, including hallucinations, depression, anxiety, thought and obsessive verbalization, physical agitation, and motor behavior without purpose, some of them of high prevalence in the DBL. These CBs seem to have conditioned the therapeutic approach of the case, but have been controlled only through the pharmacological adjustment made. Despite the success of a more comprehensive approach in the gerontological center (Table 1), some persistent CBs are maintained in terms of ideation, proactive verbalization of sexual content, and daytime sleepiness, in addition to CBs related to food.

### 4.3. Pharmacological and Non-Pharmacological Approach to CBs

The pharmacological treatment of dementias implies an etiological and symptomatic perspective. It is based on drugs that act on cholinergic systems and the competitive antagonism of *N*-methyl-d-aspartate receptors to cope with cognitive impairment and BPSD, and a wide range of psychotropic drugs for the control of BPSD, including antipsychotics, antidepressants, and benzodiazepines, among others [22,23,24,25,26]. There are good practice guidelines that question their use as chemical restraints and suggest specific time restrictions [27]. In the case we present, we believe that hypersensitivity to the use of neuroleptics is evident (a criterion suggestive of DBL), in addition to a certain therapeutic anarchy, with the use of different drug options and a very heterogeneous response. The pharmacological management of the case was redirected by understanding the disease as a symptomatological cluster, which led to the use of fluvoxamine for the control of obsessive symptoms and the use of valproate as mood stabilizer, associated with clorazepate for anxiety and rivastigmine for cognitive stabilization, with highly satisfactory results.

Regarding non-pharmacological interventions, there are different experiences that suggest the efficacy of individual and group intervention programs with the use of reminiscence and music therapy techniques, combined with behavioral interventions and environmental adaptations [26,28,29]. In our case, we believe that the modification of the habitual living space contributed positively to the control and positive evolution of the resident. The methodology suggested by Facal et al. [8] was used in the sense of an algorithmic approach from the understanding of the needs of the person, with stimulatory and therapeutic activities based on the individualization of the tasks and the direct observation of the responses, taking into account preferences and possibilities of successful performance, working actively in the prevention of CBs through the joint elaboration of narratives that give meaning to the experience in the center, promoting the active involvement and respecting the user preferences. The formative and socio-educational intervention in the relatives of the users as co-participants of the interventions, and care programs were also important. This implies considerations at an environmental level. The training and guidance of staff in the management of CBs, combining technical knowledge with group work techniques, psychological and educational support, emotional support focused on empathy, recognition of feelings and efforts, and, finally, a comprehensive institutional support system is considered essential [30,31,32].

## 5. Conclusions

In this paper, we illustrate the use of the terms BPSD, for clinical contexts, and CB, for care contexts, in order to describe the case of a 68-year-old institutionalized individual, with an initial diagnosis of LBD. Pharmacological treatment, including treatment with rivastigmine, clorazepate, valproate, and fluvoxamine, and the development of a comprehensive dementia care program within the gerontological center, led to behavioral and cognitive improvements. According to these improvements and the presence of obsessive and depressive symptoms, the diagnosis of a degenerative dementia is discussed. 

Implications of an adequate pharmacological and environmental management are discussed, considering these approaches central in the care of users with CBs in geriatric and gerontological centers. Modifications in the consideration of the disease as a heterogeneous symptomatological cluster, and in the management of environmental issues within a comprehensive care program, are considered the key aspects of a partially successful treatment.

## Figures and Tables

**Table 1:** Testological evaluation and evolutionary pharmacological treatment.

30/01/2016	31/01/2017
**Test**	**Test**
SMMSE: 23	SMMSE: 30
MMSE: 20	MMSE: 23
APADEM-NH: 46	APADEM-NH: 18
CORNELL: 18	CORNELL: 13
NPI: 58	NPI: 9
CMAI: 84	CMAI: 53
CDR: 3	CDR: 2
**Drugs**	**Drugs**
Rivastigmine = 9.5 mg/24 h	Rivastigmine = 9.5 mg/24 h
Olanzapine = 10 mg/24 h	Fluvoxamine = 100 mg/24 h
Valproate Sodium = 1000 mg/24 h	Valproate Sodium = 1000 mg/24 h
Clorazepate = 45 mg/24 h	Clorazepate = 15 mg/24 h

Note: MMSE = Mini-Mental State Examination, SMMSE = Severe Mini-Mental State Examination, CSDD = Cornell’s Scale for Depression in Dementia, NPI-NH = Neuropsychiatric Inventory—Nursing Home, CMAI = Cohen-Mansfield Agitation Inventory, APADEM-NH = Apathy in Dementia—Nursing Home, and CDR = Clinical Dementia Rating.

**Table 2:** Evolution in the MMSE score.

30/01/2016	24/02/2016	24/08/2016	2/03/2017	6/09/2017	21/03/2018	3/10/2018
20	11	23	19	22	23	25
